# Distinct Roles for Hematopoietic and Extra-Hematopoietic Sphingosine Kinase-1 in Inflammatory Bowel Disease

**DOI:** 10.1371/journal.pone.0113998

**Published:** 2014-12-02

**Authors:** Ashley J. Snider, Wahida H. Ali, Jonathan A. Sticca, Nicolas Coant, Amr M. Ghaleb, Toshihiko Kawamori, Vincent W. Yang, Yusuf A. Hannun, Lina M. Obeid

**Affiliations:** 1 Northport Veterans Affairs Medical Center, Northport, New York, United States of America; 2 Department of Medicine, Stony Brook University, Stony Brook, New York, United States of America; 3 Stony Brook Cancer Center, Stony Brook University, Stony Brook, New York, United States of America; 4 University of South Carolina, School of Medicine, Columbia, South Carolina, United States of America; 5 University of Hawaii Cancer Center, Honolulu, Hawaii, United States of America; 6 Department of Pathology, Ichinomiya Nishi Hospital, Aichi, Japan; Charité-Universitätsmedizin Berlin, Germany

## Abstract

Sphingosine kinase 1 (SK1), one of two SK enzymes, is highly regulated and has been shown to act as a focal point for the action of many growth factors and cytokines. SK1 leads to generation of sphingosine-1-phosphate (S1P) and potentially the activation of S1P receptors to mediate biologic effects. Our previous studies implicated SK1/S1P in the regulation of inflammatory processes, specifically in inflammatory bowel disease (IBD). These studies were conducted using a total body knockout mouse for SK1 and were unable to determine the source of SK1/S1P (hematopoietic or extra-hematopoietic) involved in the inflammatory responses. Therefore, bone marrow transplants were performed with wild-type (WT) and SK1-/- mice and colitis induced with dextran sulfate sodium (DSS). Irrespective of the source of SK1/S1P, bone marrow or tissue, DSS induced colitis in all mice; however, mice lacking SK1 in both hematopoietic and extra-hematopoietic compartments exhibited decreased crypt damage. Systemic inflammation was assessed, and mice with WT bone marrow demonstrated significant neutrophilia in response to DSS. In the local inflammatory response, mice lacking SK1/S1P in either bone marrow or tissue exhibited decreased induction of cytokines and less activation of STAT3 (signal transducer and activator of transcription 3). Interestingly, we determined that extra-hematopoietic SK1 is necessary for the induction of cyclooxygenase 2 (COX2) in colon epithelium in response to DSS-induced colitis. Taken together our data suggest that hematopoietic-derived SK1/S1P regulates specific aspects of the systemic inflammatory response, while extra-hematopoietic SK1 in the colon epithelium is necessary for the autocrine induction of COX2 in DSS-induced colitis.

## Introduction

Sphingolipids, originally described as a family of lipids with structural roles, are emerging as potent bioactive lipids with distinct biological functions. Three key bioactive sphingolipids have been extensively studied; ceramide and sphingosine are implicated in cell death [1]–[3], growth arrest and senescence [4], [5], while S1P is associated with proliferation [6], migration and cytoskeletal rearrangement [7], [8]. Of recent interest is the role for S1P and its synthetic enzyme, SK1 in inflammation and immune cell trafficking. S1P binds to a family of five G-protein coupled receptors, namely S1PRs to elicit many of its effects; although novel intracellular targets have also recently been identified. Studies using the S1PR agonist FTY720, which down-regulates S1PRs and induces lymphopenia, implicate the binding of S1P to its receptors in immune cell trafficking [9]. In contrast, SK1 is implicated in the function of immune cells including cytokine production and chemotaxis in macrophages [10], oxidative burst and migration in neutrophils [11], and mast cell degranulation and migration [12], [13].

SK1 is highly regulated and has been shown to be activated by pro-inflammatory cytokines such as interleukin1 beta (IL1β) and tumor necrosis factor alpha (TNFα) both *in vitro* [14], [15] and *in vivo* [16]. This activation by cytokines leads to induction of COX2 and production of prostaglandins [14]. We have previously demonstrated that this pathway also occurs *in vivo* in an animal model of DSS-induced colitis and TNF-induced arthritis and that loss of SK1 prevents COX2 induction in both of these murine models of inflammation [16], [17].

There are two subclasses of IBD: ulcerative colitis (UC) and Crohn's Disease (CD). UC is restricted to mucosal inflammation in the colon, whereas CD can affect the entire gastrointestinal tract. Immunosuppressive agents have classically been utilized for the treatment of IBD; however, recent therapies have been developed to target more specific mediators of IBD, such as the increases in circulating and tissue TNFα. Previous studies, by our group and others, have demonstrated that pro-inflammatory cytokines such as TNFα can activate SK1 in cells and *in vivo* [14]–[17]. Furthermore, we showed that total body genetic deletion of SK1 partially protected mice from DSS-induced colitis [16]. Interestingly, genetic deletion of SK2 highlights opposing functions for the SK isoforms as loss of SK2 in both IBD [18] and rheumatoid arthritis (RA) [19] has been demonstrated to provide no protection from, and potentially worsen, inflammation or disease.

Our previous study demonstrated an increase in SK1 expression in colon tissue from patients with ulcerative colitis [16]. In addition, we observed an increase in SK1 expression and activity in colon tissue in DSS treated WT mice, as well as, an increase in S1P in circulation. SK1^−/−^ DSS-treated mice were protected from both systemic inflammation and the local inflammatory response in colon tissue. However, by using the total body knockout we were unable to determine if tissue SK1/S1P were influencing the inflammatory response or if these factors were more important in the hematopoietic-derived cell population. In the current study we utilized bone marrow transplanted mice to determine the roles of each of these cell populations in DSS-induced colitis. Here we demonstrate distinct roles for SK1/S1P in the local and systemic inflammatory responses; where hematopoietic-derived cells is critical for neutrophilia and extra-hematopoietic SK1 in colon epithelium is necessary for the COX2 expression in response to DSS-induced colitis.

## Materials and Methods

### Mice

C57BL/6 WT mice were purchased from Charles River Laboratories (Wilmington, MA, USA) and sphingosine kinase-1 knockout mice (SK1^−/−^) mice from Dr. Rick Proia were backcrossed at least six generations to C57BL/6. Animals were maintained under standard laboratory conditions. All animal procedures were approved by the Ralph H. Johnson VA Medical Center Institutional Animal Care and Use Committee and followed the guidelines of the American Veterinary Medical Association.

### Bone marrow transplant

Six to eight old male WT and SK1^−/−^ C57BL/6 mice were used as donor mice for the bone marrow transplant, whereby mice were euthanized and bone marrow collected. Briefly, bone marrow collected by flushing the femur with HBSS, bone marrow cells counted using crystal violet and finally resuspended in PBS for injection into irradiated recipient mice. Ten to twelve week old female WT and SK1^−/−^ recipient mice were sub-lethally irradiated using 1100 rads in two doses (2×550 rads) from a ^137^Cs irradiator. Following irradiation, 2×10^6^ bone marrow cells were injected into the tail vein of recipient mice. Mice were maintained on 1 mg/ml neomycin in their drinking water for 2 weeks and then provided with regular drinking water for 4 weeks of additional recovery.

### Induction of colitis

Acute colitis was induced by adding 5% (w/v) DSS (MP Biomedicals, Inc., Solon, OH, USA) to drinking water for 5 days. DSS solutions were monitored to ensure equal consumption among the groups of mice. Untreated mice were given regular drinking water.

### Histology score

Colons were removed, rinsed with PBS, opened longitudinally, and fixed with 10% formalin. Sections were embedded in paraffin, fixed to glass slides, and stained with hematoxylin and eosin (H&E). The entire colon was microscopically examined for damage, and scored in a blinded fashion. Scores were assigned as previously described [20]; briefly sections were scored based on inflammation severity (1–3), inflammation extent (1–3) and crypt damage (1–4) and the score was multiplied by the percent involvement resulting in a minimal score of 0 and a maximal score of 40.

### Lipid analysis

Advanced analyses of sphingosine and ceramide species in whole blood were performed by the Lipidomics Core at MUSC on a Thermo Finnigan TSQ 7000, triple-stage quadrupole mass spectrometer operating in a Multiple Reaction Monitoring (MRM) positive ionization mode as described [21].

### Harvesting of RNA

Colon tissue was collected from the formalin fixed and embedded in paraffin sections and RNA was isolated using a FFPE RNeasy (Qiagen, Valencia, CA, USA) per the manufacturer's instructions. RNA yield and purity were assessed by spectroscopic analysis.

### Real-time RT-PCR

RNA was extracted from formalin-fixed paraffin embedded (FFPE) tissues using the Qiagen FFPE RNeasy kit according to the manufacturer's instructions. RNA was reverse transcribed into cDNA using 0.5 µg RNA, Oligo_DT_ primers and SuperScriptIII from Invitrogen (Carlsbad, CA, USA). Real-time RT-PCR was performed on an ABI 7500 to quantify mRNA levels of IL1β, TNFα, and IL-6. The standard real-time RT-PCR reaction volume was 20µl, including 10µl iTaq Universal Probe master mix PCR reagents (Biorad, Hercules, CA, USA), 4µl cDNA template, 1µl TaqMan Gene Expression Assay primers, and 5µl water. The RT-PCR steps were as follows: 2 minutes at 95°C, followed by cycles (*n* = 40) consisting of 15 s melt at 95°C, 60 s annealing/extension at 60°C and a final step of 1 min incubation at 60°C. All reactions were performed in triplicate. The data were analyzed using Q-Gene software [22] and expressed as mean normalized expression (MNE). MNE is directly proportional to the amount of RNA of the target gene relative to the amount of RNA of the reference gene β-actin.

### Immunohistochemistry

Phosphorylation of STAT3 and expression of COX2 were assessed by immunohistochemistry (IHC). Additionally, the presence of macrophages and neutrophils were assessed. Briefly, sections were deparaffinized and rehydrated with serial ethanol dilutions. Endogenous peroxidase activity was neutralized using sodium periodate (0.005 M pH2.5) and sodium borohydrate (0.003 M). Antigen retrieval was performed using citrate buffer (pH 6). After washing in PBS, sections were blocked for 20 minutes with the appropriate blocking reagent from the VECTASTAIN ImmPress Kit (Vector Laboratories Inc.), which was also used for the remaining steps. Sections were then incubated for overnight at 4°C with the COX2 primary antibody (Thermo Scientific, Freemont, CA), phospho-STAT3 (Ser727, Cell Signaling, Danvers, MA), F4/80 (AbD Serotec, Raleigh, NC), or GR1-Ly6 (BD Pharmingen, San Jose, CA) followed washing with PBS and 30 min incubation with biotinylated secondary antibody and incubation with DAB reagent. Slides were lightly counterstained with hematoxylin, rehydrated, and mounted for analysis by bright field microscopy.

### Data analysis

Statistical analyses were performed using a Two-way ANOVA to test for species and treatment effects, and one-way ANOVA for analysis among treated groups. A *p* value below 0.05 was considered significant. Significance for each figure is detailed in the figure legends.

## Results

### Loss of SK1 in hematopoietic-derived cells does not prevent parameters of colitis

**Figure 1:**
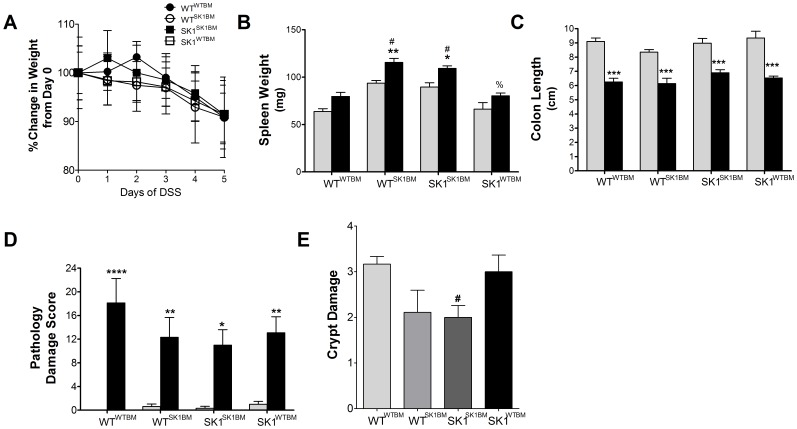
Mice with loss of SK1 in hematopoietic cells exhibit splenomegaly with DSS-induced colitis. Bone marrow transplants were performed with WT and SK1^−/−^ mice. Following 6 weeks of rest, mice were administered either regular drinking water or water containing 5% DSS for 5 days. **A**) Change in body weight was assessed. Data represent mean ±SD, n≥6 for each treatment group; (significance at Day 5 =  *WT^WTBM^, **WT^SK1BM^, **SK1^WTBM^); *p<0.05, **p<0.01, ***p<0.001 vs strain Day 0. **B**) Spleen weight and **C**) colon length were assessed following treatment. **D**) Pathology damage scores were determined by a pathologist in a blinded fashion with H&E sections of colon tissue. Data represent mean ±SD, n≥6 for each treatment group; *p<0.05, **p<0.01, ***p<0.001 vs strain untreated, #p<0.05 as compared to WT^WTBM^ DSS treated, %p<0.05 as compared to SK1^SK1BM^ DSS. For the X-axis: regular text refers to the host genotype and the superscript to the bone marrow genotype.

In order to determine the roles of hematopoietic and extra-hematopoietic SK1/S1P in the inflammatory responses observed with DSS-induced colitis we elected to employ bone marrow transplants in our studies. WT and SK1^−/−^ mice were sub-lethally irradiated as described in the Materials and Methods, and their bone marrow was reconstituted with either host or donor bone marrow, resulting in strain matched controls (WT^WTBM^, SK1^SK1BM^) as well as strains with the contrasting bone marrow (WT^SK1BM^, SK1^WTBM^). Following six weeks of recovery, mice were administered regular drinking water or 5% DSS for 5 days to induce acute colitis and the parameters of colitis examined. All mice that received DSS in drinking water demonstrated significant weight loss (Figure 1A), except mice lacking SK1 in both hematopoietic and extra-hematopoietic compartments. Colon shortening (Figure 1C) was observed in all treated mice; however, mice lacking SK1 in both hematopoietic and extra-hematopoietic compartments demonstrated the least colon shortening among the treated mice. Similar to what we observed in our previous study using total body SK1^−/−^ mice, untreated SK1^SK1BM^ mice demonstrated larger spleens than untreated WT^WTBM^ mice. Moreover, only mice lacking SK1 in bone marrow demonstrated significant splenomegaly with DSS treatment (Figure 1B), likely indicating that SK1 in hematopoietic-derived cells may be necessary for egress of lymphocytes from the spleen in DSS-induced colitis, as S1P has been implicated in lymphocyte egress [23].

Disease pathology was examined by a pathologist in a blinded fashion and scored as previously described [20]. All mice treated with DSS demonstrated significant increases in disease pathology (Figure 1D) as indicated by increases in immune cell infiltrate and loss of crypt structure. Overall mice lacking SK1 in bone marrow demonstrated less pathologic damage when compared to mice with WT bone marrow, and mice SK1^SK1BM^ mice demonstrated the least pathologic damage overall; however, this was not statistically significant among treatment groups. Upon examination of crypt damage, all mice demonstrated at least partial disruption of crypt structure (Figure 1E and Figure S1); however, SK1^SK1BM^ mice exhibited statistically less crypt damage when compared to WT^WTBM^. Together these data suggest that loss of SK1 confers some protection in DSS-induced colitis; however, either the procedure of irradiation and/or bone marrow transplant may sensitize mice to DSS-induced colitis.

### Hematopoietic-derived SK1 regulates sphingolipid levels in circulation

**Figure 2:**
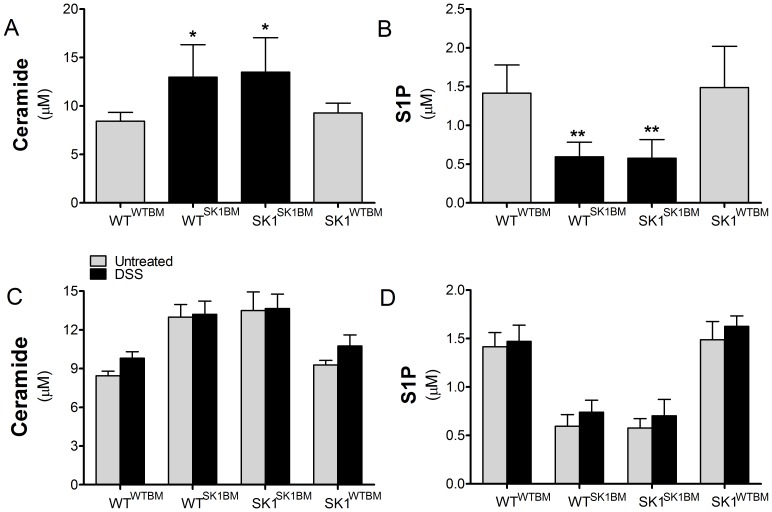
Hematopoietic genotype determines circulating sphingolipid levels. Whole blood was collected and analyzed for sphingolipid content using ESI/MS/MS. **A and C**) Ceramide and **B and D**) S1P levels were normalized to volume. Data represent mean ±SD, n≥6 for each treatment group, *p<0.05, **p<0.01 as compared to both strains of mice with WT bone marrow. X-axis: regular text refers to the host genotype and the superscript to the bone marrow genotype.

SK1 and S1P have been shown to play crucial roles in immune cell trafficking. In addition, we have demonstrated previously that S1P in circulation increases with DSS-induced colitis and that this may play a role in the systemic inflammatory response [16], [24]. In order to determine if S1P levels in circulation could be affecting spleen size and/or systemic inflammation, we first measured circulating sphingolipids. Whole blood was collected, and sphingolipids were analyzed by LC-MS/MS. Mice with intact SK1 in bone marrow-derived cells, WT^WTBM^ and SK1^WTBM^, exhibited significantly higher circulating S1P and significantly lower levels of ceramide when compared to mice deficient in hematopoietic SK1 (Figure 2A and B). Examination of specific ceramide species revealed that WT^SK1BM^ and SK1^SK1BM^ demonstrated significantly higher levels of C24 ceramide when compared to mice with WT^WTBM^ and SK1^WTBM^ mice (Table S1). In contrast to our previous study, there were slight changes in S1P and major ceramide species in circulation following DSS-induced colitis, but none of these reached significance (Figure 2C and D). In WT^WTBM^ mice S1P increased from 1.41±0.36 µM in untreated mice to 1.47±0.36 µM in DSS treated mice, and from 0.59±0.34 µM to 0.739±0.35 µM in WT^SK1BM^ mice. Similarly, S1P increased from 0.57±0.24 µM to 0.71±0.42 µM in SK1^SK1BM^ mice and 1.48±0.53 µM to 1.62±0.34 µM in SK1^WTBM^ mice upon DSS treatment. These data demonstrate that hematopoietic-derived SK1 is a major contributor to circulating S1P and regulates circulating ceramide levels.

### Hematopoietic SK1 regulates systemic inflammation following DSS-induced colitis

**Figure 3:**
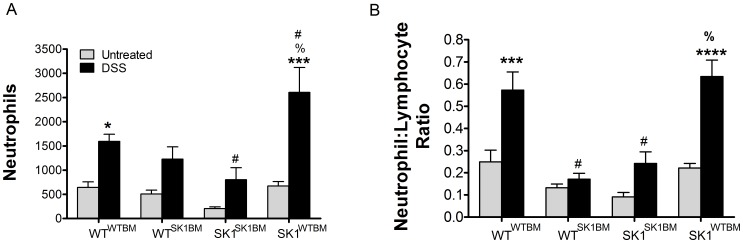
Mice with WT bone marrow exhibit significant neutrophilia following DSS-induced colitis. **A**) Whole blood was collected and analyzed for neutrophil counts. **B**) Neutrophil counts are normalized to total lymphocytes and expressed as neutrophil-lymphocyte ratio. Data represent mean ±SD, n≥6 for each treatment group; *p<0.05, ***p<0.001 and ****p<0.001 vs strain untreated, #p<0.05 as compared to WT^WTBM^ DSS treated, %p<0.05 as compared to SK1^SK1BM^ DSS. X-axis: regular text refers to the host genotype and the superscript to the bone marrow genotype.

Increases in circulating lymphocytes have been shown to be indicative of systemic inflammation in colitis; however, recently our data and others imply that the number of circulating neutrophils may be a reliable indicator of active colitis [25], [26]. In order to determine if hematopoietic SK1 influences systemic inflammation in DSS-induced colitis, we collected blood samples and examined complete blood counts (CBCs) from bone marrow transplanted mice. There were no significant differences in CBCs among any of the untreated mice, indicating that neither irradiation nor the bone marrow transplantation affected hematopoietic-derived cell populations. Following DSS treatment, all mice demonstrated increases in circulating white blood cells (WBCs) and loss of red bloods cells (RBCs) (Table S2). An increase in WBCs is indicative of systemic inflammation and loss of RBCs is indicative of bleeding into the GI lumen suggesting that all mice were at least partially affected by the DSS. However, the most remarkable difference among treatments groups was observed in neutrophil counts in mice with WT bone marrow. As shown in Figure 3A, WT^WTBM^ and SK1^WTBM^ mice exhibited significant increases in circulating neutrophils following DSS administration. Moreover, the neutrophil-lymphocyte ratio, which is emerging as a marker for active disease in human patients [25], [26], was significantly elevated in WT^WTBM^ and SK1^WTBM^ mice (Figure 3B). These data suggest that SK1/S1P in hematopoietic-derived cells is important for recruitment of neutrophils into the circulation.

Of note, our previous study demonstrated neutrophil recruitment into colon tissue was at least partially dependent on SK1 [16]. In this study we observed decreased neutrophils in the colon from mice lacking SK1 in either the bone marrow or tissues as compared to WT^WTBM^ mice (Table S3). These data suggest that SK1 is potentially important in both hematopoietic and non-hematopoietic cells for neutrophil recruitment in the local inflammatory response.

### Local inflammatory responses require extra-hematopoietic SK1

**Figure 4:**
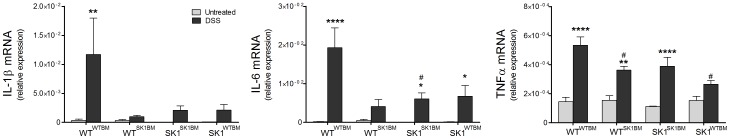
Dual sources of SK1/S1P regulate IL-1β and IL-6 expression in DSS-treated mice. Expression levels of **A**) IL-1β, **B**) IL-6, and **C**) TNFα in colon tissues were determined using real time-RTPCR and normalized to β-actin. Data represent mean ±SEM, n≥3 for each treatment group; *p<0.05, **p<0.01, and ****p<0.001 vs strain untreated, #p<0.05 as compared to WT^WTBM^ DSS. X-axis: regular text refers to the host genotype and the superscript to the bone marrow genotype.

Induction of pro-inflammatory cytokines and pro-inflammatory mediators such as COX2 and STAT3 in colon tissue have been demonstrated in animal models of colitis and colitis associated cancer (CAC). Our previous results in a total body knockout for SK1 demonstrated that SK^−/−^ mice with DSS-induced colitis exhibited an exaggerated TNFα response but failed to induce COX2 in colon tissue. Moreover, we observed that WT mice but not SK^−/−^ mice induced activation (translocation) of STAT3 (Figure S2). Others have also recently demonstrated that SK1 is required for phosphorylation of STAT3 in CAC [18]. Therefore, in our bone marrow transplant model we sought to determine which source of SK1/S1P was necessary for the induction of pro-inflammatory mediators and downstream signaling events in the colon. To this end, IL-1β, IL-6, and TNFα expression levels were examined in colon tissue from bone marrow transplanted mice. IL1β expression levels increased in all treatment groups; however, this was only statistically significant in WT^WTBM^ mice following DSS-induced colitis (Figure 4A). IL-6 and TNFα expression levels were increased in all treatment groups following DSS-induced colitis (Figure 4B & C); however, the largest increases were observed in WT^WTBM^ mice. The deletion of SK1 from either source resulted in strikingly less IL-1β and IL-6 expression, but not TNFα, upon DSS treatment. Of note, we examined nuclear localization of nuclear factor kappa B (NFκB) in response to DSS in all treatment groups. Despite the differences in cytokine expression there were no significant differences in NFκB localization (data not shown). These data suggest that loss of SK1 in either the hematopoietic or extra-hematopoietic cell populations decreases specific pro-inflammatory cytokines in the colon; thus indicating potential dual sources of SK1/S1P necessary for pro-inflammatory cytokines in response to DSS-treatment.

**Figure 5:**
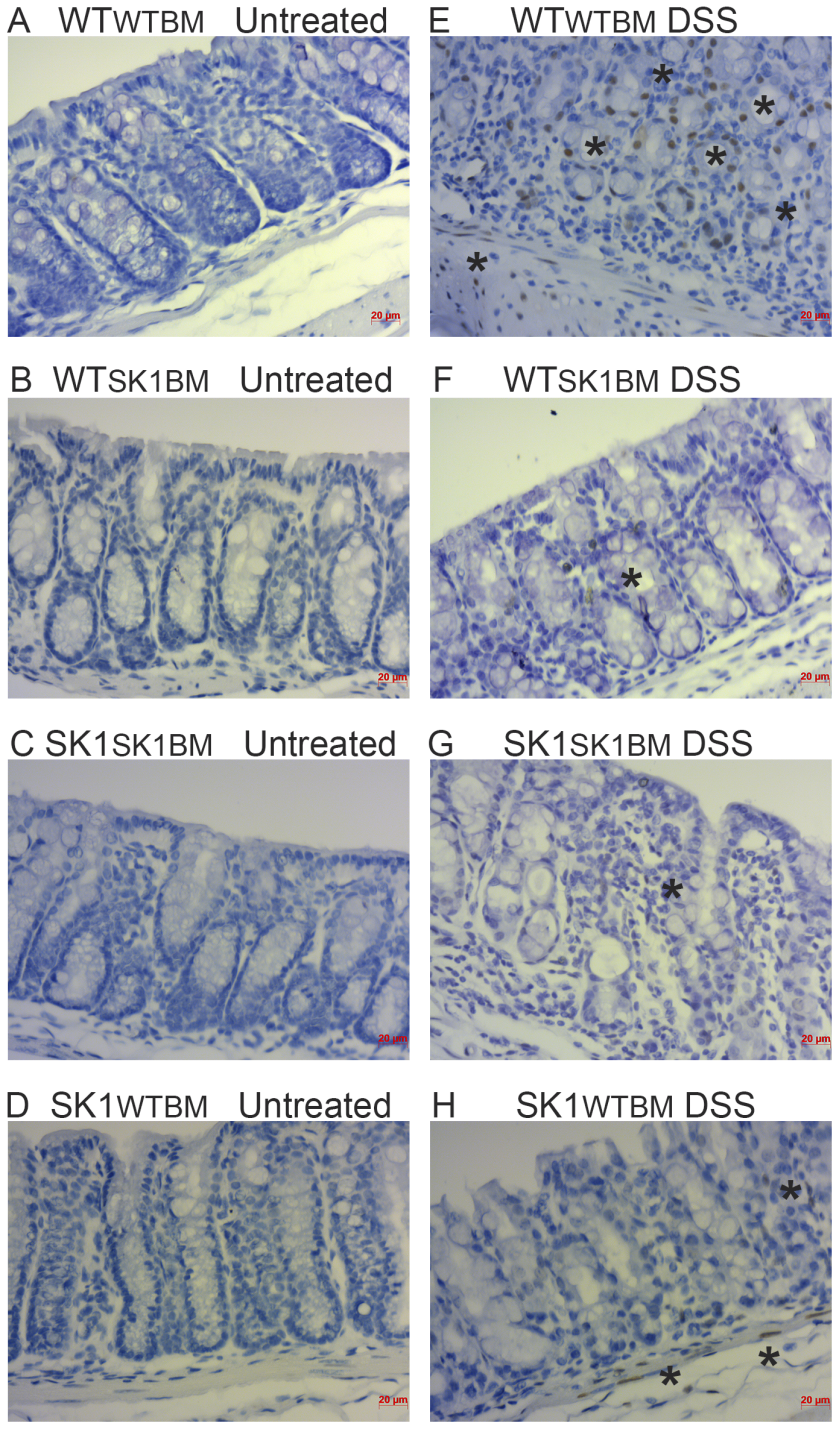
STAT3 phosphorylation requires dual sources of SK1/S1P in acute DSS-induced colitis. Phospho-STAT3 (Ser727) was examined by IHC. **A–D**) untreated mice; **E–H**) DSS treated mice. Scale bars  = 20 µm. Regular text refers to the host genotype and the superscript to the bone marrow genotype.

Binding of IL-6 to its receptor results in activating phosphorylation of the transcription factor STAT3, which has been implicated downstream of S1P and S1PR1 [18]. IHC analysis of colon tissues revealed that upon DSS treatment mice lacking SK1 in either compartment, hematopoietic or extra-hematopoietic, demonstrated lower expression of phospho-STAT3 (Figure 5F, G, & H). Similar to IL-6 expression, all mice treated with DSS demonstrated some activation of STAT3, including SK1^SK1BM^ mice; however, all mice lacking SK1 from either bone marrow or tissue exhibited reduced phospho-STAT3 as compared to WT^WTBM^ mice (Table S3). These data suggest that STAT3 phosphorylation is partially dependent on SK1 in both hematopoietic and extra-hematopoietic cells, but that SK1 is not required for phosphorylation of STAT3 in an acute colitis model.

**Figure 6:**
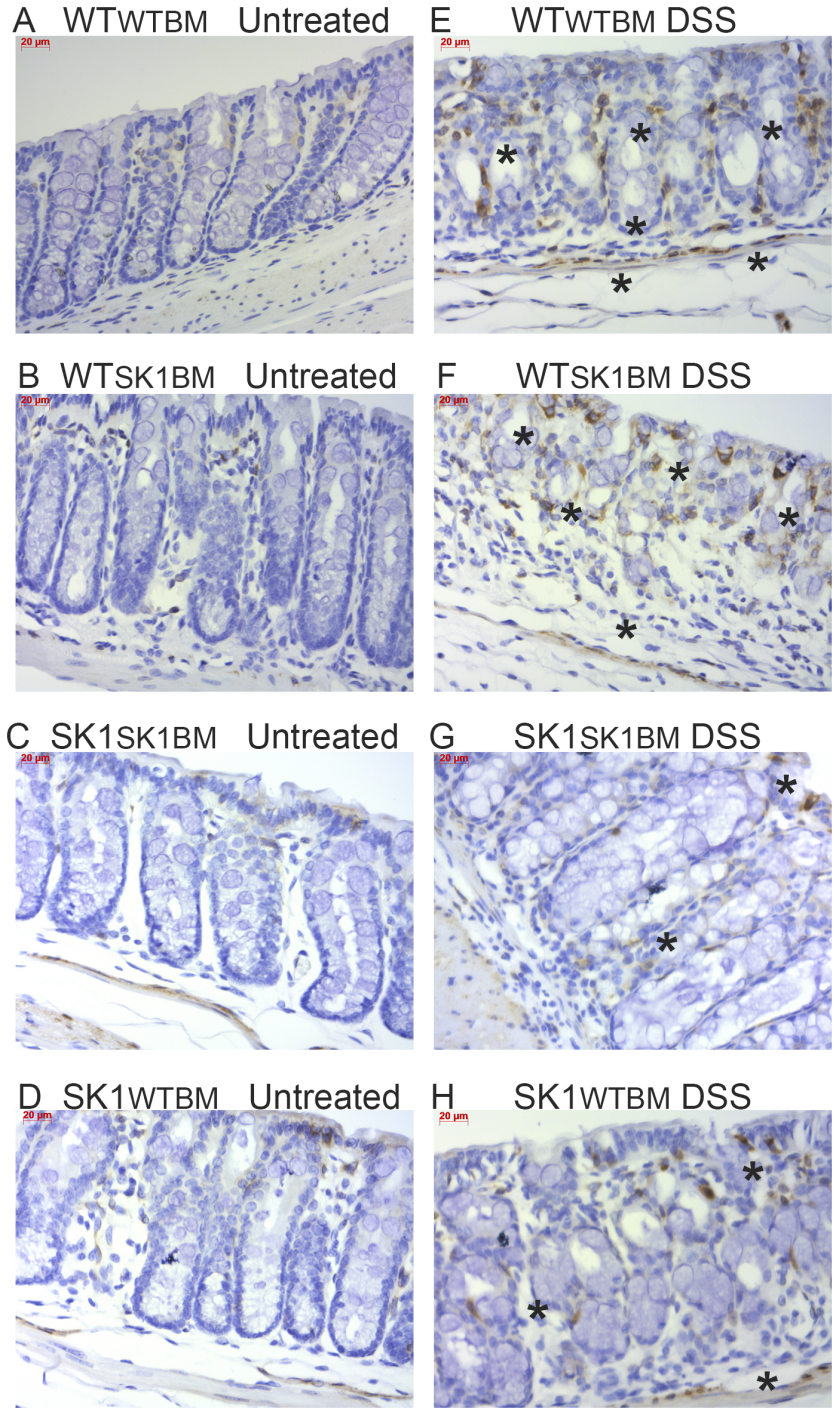
Extra-hematopoietic SK1 is necessary for COX2 expression in the colon epithelium. COX2 expression levels were examined by IHC **A–D**): untreated mice; **E–H**) DSS treated mice. Scale bars  = 20 µm. Regular text refers to the host genotype and the superscript to the bone marrow genotype.

Additionally, we examined COX2 expression in colon tissues following DSS-induced colitis. Expression of COX2 significantly increased in WT^WTBM^ following DSS administration (Figure 6E) and SK1^SK1BM^ mice demonstrated no discernable induction of COX2 with DSS-induced colitis (Figure 6G) recapitulating the lack of COX2 expression in SK^−/−^ that we had observed in our previous study. The most interesting changes in COX2 expression following DSS-induced colitis occurred in WT^SK1BM^ mice (Figure 6F), which demonstrated significant increases in epithelial COX2 expression. On the other hand, SK1^WTBM^ mice demonstrated slight COX2 expression, which was much less than WT^SK1BM^ mice. These data demonstrate that extra-hematopoietic SK1 is primarily responsible for COX2 expression in the colonic epithelium following DSS-induced colitis.

## Discussion

Sphingosine kinase 1 and S1P have been shown to play key roles in numerous inflammatory processes and disease states. Our previous results demonstrated that total body SK1^−/−^ mice were partially protected from DSS-induced colitis [16]. In this study we sought to determine the source of SK1/S1P necessary to mediate the inflammatory responses in this disease model using bone marrow transplants. In this study we demonstrate that mice lacking SK1 in both the hematopoietic and extra-hematopoietic compartments are protected from weight loss, as demonstrated previously [16], as well as crypt damage in response to DSS. Furthermore, we demonstrate mice with WT bone marrow exhibited significantly more neutrophilia following DSS-induced colitis, suggesting that hematopoietic-derived SK1/S1P are necessary for the recruitment of specific immune cells in circulation and are a critical part of the systemic inflammatory response. Our data also demonstrate that specific cytokines, as well as STAT3 activation, require SK1/S1P from both hematopoietic and extra-hematopoietic sources in acute DSS-induced colitis. Additionally, we show here that extra-hematopoietic SK1/S1P is necessary for the induction of COX2 in the colon epithelium in response to DSS. Taken together these data suggest that SK1/S1P play critical and distinct roles in both systemic and local inflammatory responses in DSS-induced colitis.

In this study we observed a basal increase in spleen size in mice lacking hematopoietic SK1, irrespective of tissue genotype. Moreover, both SK1^SK1BM^ and WT^SK1BM^ mice demonstrated larger spleens following DSS. Previously we demonstrated that total body loss of SK1 increased basal spleen weight; however, mice lacking SK1 failed to exhibit splenomegaly as WT mice did when challenged with DSS [16]. Interestingly, several studies in lymphocyte egress and trafficking have also demonstrated that hematopoietic sources of S1P are necessary for egress of lymphocytes from the spleen in mice deficient in both isoforms of SK [27]. Furthermore, mice deficient in the S1P degrading enzyme S1P lyase (S1PL) have demonstrated decreased cellularity in the spleen [28], consistent with our results on the influence of circulating S1P on lymphocyte trafficking from the spleen. Interestingly, we have examined the cellular composition of both WT and SK1^−/−^ mice using FACs analysis and found no differences in total macrophage, neutrophil, B or T cell numbers (data not shown). Taken together, these data further suggest that SK/S1P in hematopoietic-derived cells, and specifically SK1, may play a critical role in spleen cellularity and size.

Deletion of SK1 in hematopoietic cells resulted in a 50% reduction in total circulating S1P irrespective of tissue genotype, similar to what we and others have observed in total body SK1 knockout mice [16], [29]. As mentioned previously this loss of SK1/S1P results in basal changes in spleen size; however, there were no significant differences basally in circulating immune cells. Upon DSS treatment, mice with WT bone marrow showed significant increases in circulating neutrophils, whereas mice deficient in bone marrow SK1 showed slight but insignificant increase in circulating neutrophil counts indicating that a hematopoietic SK1/S1P are likely required for neutrophilia in response to inflammatory stimuli. Of note, neutrophils from SK1^−/−^ mice have been shown to function normally [30], [31], but SK/S1P have been shown to act as a chemoattractants for neutrophils both *in vitro* and *in vivo* [32]. Likewise, we have previously demonstrated that circulating S1P levels can influence circulating neutrophil counts, specifically in the DSS model of colitis [24]. Moreover, others have shown that mice lacking S1PL have increased S1P concentrations in circulation and this results in dramatic increases in circulating neutrophils [28],[33]. Taken together, these data suggest for the first time in an *in vivo* model of disease that circulating S1P can regulate circulating neutrophil levels and that this S1P is derived from hematopoietic SK1.

Importantly, increases in circulating neutrophil counts are beginning to be utilized as important clinical diagnostics, as recent implications have suggested that not only are neutrophils in circulation indicative of systemic inflammation, but that the ratio of neutrophils to lymphocytes in circulation may be more indicative of active colitis [25], [26]. In Figure 3B, our data demonstrate that mice with SK1 present in bone marrow exhibit significant increases in neutrophil-lymphocyte ratio. These data suggest that S1P levels in circulation directly influence neutrophilia indicating that S1P could potentially serve as a diagnostic indicator of active colitis and/or other inflammatory diseases.

Induction of pro-inflammatory cytokines in colitis is often associated with severity of disease. Indeed, anti-TNFα therapies are heavily utilized to treat patients with IBD. It has been demonstrated that TNFα, as well as other cytokines (IL-1β and IL-6) are elevated in colon tissue in response to DSS-induced colitis by our group and countless others. Similarly, SK1/S1P have been implicated both upstream [34] and downstream [14], [15], [35] of cytokine production. Here we demonstrate that both hematopoietic and extra-hematopoietic-derived SK1/S1P modulate the expression of IL-1β and IL-6, but not TNFα, suggesting dual sources of SK1/S1P in the induction of pro-inflammatory cytokines.

STAT3, a tightly regulated transcription factor, is activated upon phosphorylation in response to IL-6 and has been shown to be present in a number of cancers. SK1 has recently been suggested to be important in the activation of STAT3 in acute colitis (our data in Fig. S1) and in chronic colitis associated cancer models [18]. Here we demonstrate that loss of SK1 from either source results in decreased STAT3 phosphorylation upon acute DSS treatment, indicating the importance of SK1 for STAT3 activation in short term models of colitis as well. We have recently demonstrated that SK1 is required for IL6 production in skeletal muscle cells in response to fatty acid stimulation [36]. Therefore, it is possible that SK1 regulation of IL6 in colon tissues (Figure 4B) indirectly prevents STAT3 activation by modulation of IL6. These data suggest that both hematopoietic and extra-hematopoietic sources of SK1/S1P are important in phosphorylation of STAT3 in an acute animal model of disease.

COX2 has been shown to play a critical role in colon tissue responses in inflammation and in cancer. We and others have used COX2 induction as a marker for inflammation and disease severity in animal models of colitis [16], [24], [37], [38]. Furthermore, we have demonstrated *in vivo* that SK1 is required for COX2 induction in arthritis, colitis, and CAC [16], [17], [39]. Our current study demonstrates an essential role for extra-hematopoietic SK1 in induction of COX2 in the colon epithelium. In our previous study total body SK1 knockout mice challenged with DSS were protected from colitis, at least partially due to this inability to induce COX2 in the colon [16]. We have also demonstrated significant increases in S1P levels in the colon epithelium in response to DSS, this also corresponded with a significant increase in COX2 expression at the mRNA level [24]. Taken together our data demonstrate that SK1 and autocrine S1P are necessary for COX2 induction in colon epithelium; suggesting perhaps inhibition of SK1, as an upstream inducer of COX2, in colon epithelial cells may prove to be a valid therapeutic target, as well as provide more insight into the role for SK1 and COX2 in IBD.

In summary, our data demonstrate a critical role for hematopoietic-derived SK1 in circulating S1P levels, as well as systemic immune responses, specifically neutrophilia. In addition, these data illustrate that dual sources of SK1/S1P regulate the induction of pro-inflammatory cytokines and STAT3 activation. Finally, our data demonstrate that extra-hematopoietic SK1 is required for induction of COX2 in colon tissue in response to DSS-induced colitis. Future studies will examine the potential for SK1 as a therapeutic target in in the inflammatory processes involved with IBD.

## Supporting Information

Figure S1 **H&E sections from bone marrow transplant mice.** Tissue sections from bone marrow transplanted mice were stained using H&E. Representative sections from **A–D**) untreated mice; **E–H**) DSS treated mice.
(TIF)

Figure S2 **SK1 is necessary for STAT3 phosphorylation in the colon.** WT and total body SK1^−/−^ mice were colons were examined for phospho-STAT3 (Ser727) with immunofluorescence **A–B**) untreated mice; **C–D**) DSS treated mice (5% DSS for 5 days).
(TIF)

Table S1 Blood Sphingolipid Measurements. Sphingolipid levels were analyzed by ESI/MS/MS from whole blood following BMTP and treatment with or without 5% DSS for 5 days. Data are mean ±SD. *p<0.05 compared to untreated, strain-matched control, ^#^ *p*<0.05 comparison between mice of the same host genotype administered water. Regular text refers to the host genotype and the superscript to the bone marrow genotype.
(PDF)

Table S2 Complete Blood Counts. Blood counts were analyzed following BMTP and treatment with or without DSS. Data represent mean ±SD; n≥6 mice per group. ^#^ *p<0.01* compared to untreated, strain-matched control. Regular text refers to the host genotype and the superscript to the bone marrow genotype.
(PDF)

Table S3 IHC analysis of bone marrow transplant mice treated with DSS. Semi-quantitative analysis of COX2, phopsho-STAT3, macrophage (F4/80), and neutrophil (Gr-1) staining in colon tissues of bone marrow transplanted tissues following DSS treatment. Regular text refers to the host genotype and the superscript to the bone marrow genotype.
(PDF)
