# Supplementary figures and images for: Distinct Roles for Hematopoietic and Extra-Hematopoietic Sphingosine Kinase-1 in Inflammatory Bowel Disease
Source: PLoS One. 2014 Dec 2;9(12):e113998. doi: 10.1371/journal.pone.0113998 (PMC4252067; doi:10.1371/journal.pone.0113998)

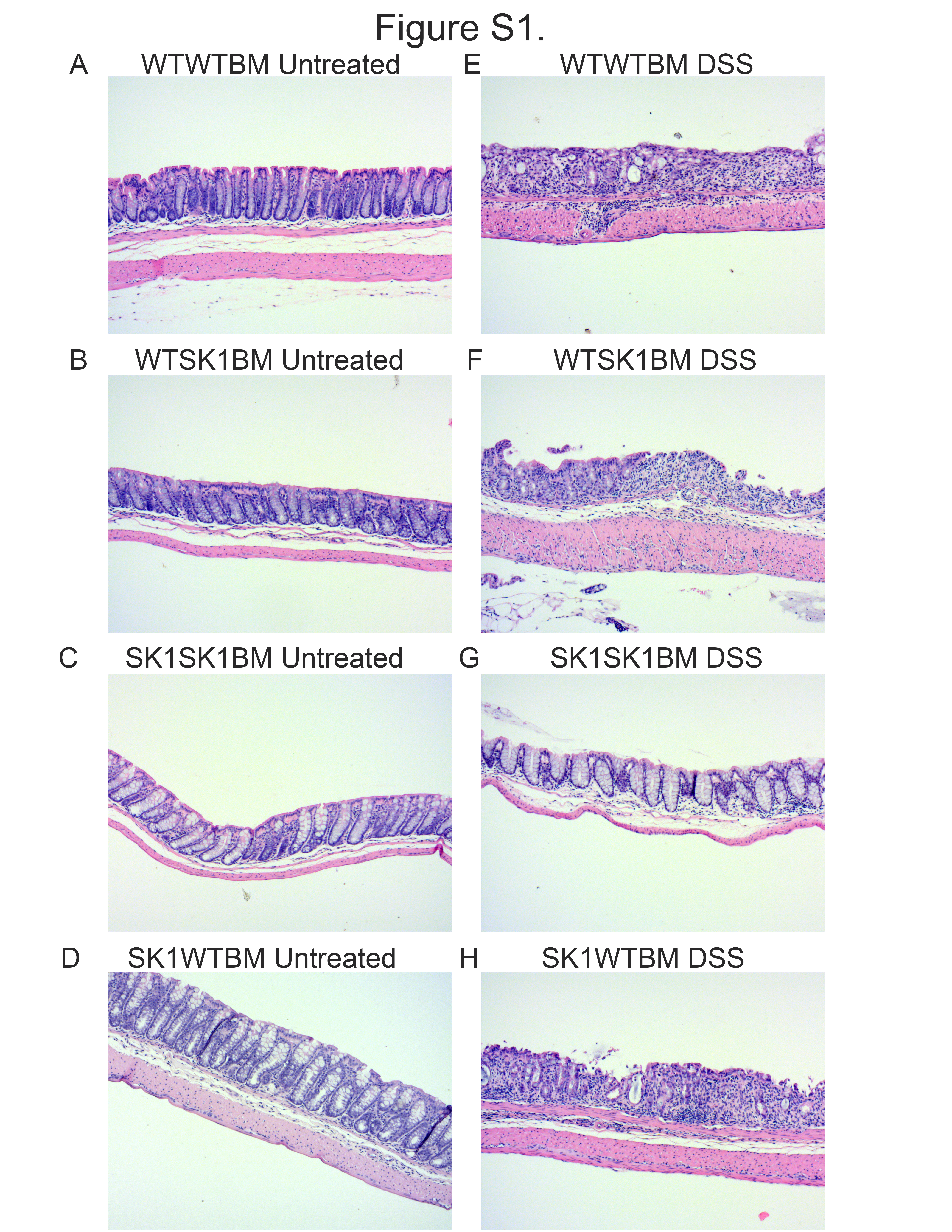

Supplement: Figure S1 — H&E sections from bone marrow transplant mice. Tissue sections from bone marrow transplanted mice were stained using H&E. Representative sections from A–D) untreated mice; E–H) DSS treated mice. (TIF) [file pone.0113998.s001.tif]

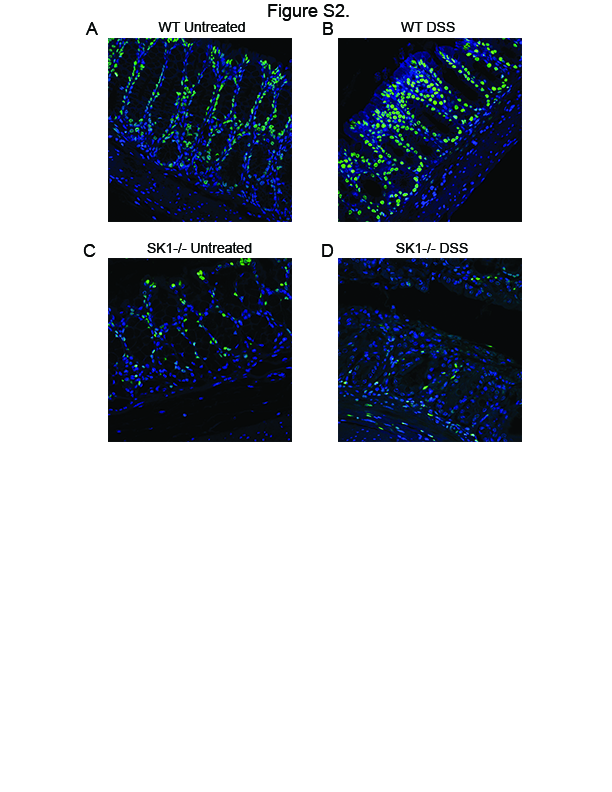

Supplement: Figure S2 — SK1 is necessary for STAT3 phosphorylation in the colon. WT and total body SK1−/− mice were colons were examined for phospho-STAT3 (Ser727) with immunofluorescence A–B) untreated mice; C–D) DSS treated mice (5% DSS for 5 days). (TIF) [file pone.0113998.s002.tif]
