# Supplementary material for: Distinct Roles for Hematopoietic and Extra-Hematopoietic Sphingosine Kinase-1 in Inflammatory Bowel Disease
Source: PLoS One. 2014 Dec 2;9(12):e113998. doi: 10.1371/journal.pone.0113998 (PMC4252067; doi:10.1371/journal.pone.0113998)
Supplement: Table S1 — Blood Sphingolipid Measurements. Sphingolipid levels were analyzed by ESI/MS/MS from whole blood following BMTP and treatment with or without 5% DSS for 5 days. Data are mean ±SD. *p<0.05 compared to untreated, strain-matched control, # p<0.05 comparison between mice of the same host genotype administered water. Regular text refers to the host genotype and the superscript to the bone marrow genotype. (PDF) [file pone.0113998.s003.pdf]

Strain	Treatment	C16 Ceramide	C24 Ceramide	Sph	Dh-Sph	Dh-S1P
WT^{WTBM}	Water	0.45 ± 0.09	4.35 ± 0.50	2.05 ± 0.87	2.79 ± 0.96	0.85 ± 0.22
WT^{WTBM}	DSS	0.53 ± 0.09	5.05 ± 0.71	3.22 ± 1.02*	4.15 ± 2.72	1.01 ± 0.33
WT^{SK1BM}	Water	0.67 ± 0.20	7.12 ± 1.34#	1.44 ± 0.76	1.48 ± 1.20#	0.26 ± 0.35#
WT^{SK1BM}	DSS	0.89 ± 0.17	6.97 ±1.99	2.16 ± 0.89	2.17 ± 1.43	0.47 ± 0.28
$SK1^{SK1BM}$	Water	0.63 ± 0.27	7.35 ± 2.11#	1.61 ± 0.23	1.43 ± 0.85#	0.27 ± .019#
$SK1^{SK1BM}$	DSS	0.76 ± 0.20	7.26 ± 1.97	1.58 ± 0.52	1.57 ± 0.77	0.46 ± 0.34
$SK1^{WTBM}$	Water	0.51 ± 0.18	4.75 ± 0.29	2.25 ± 0.73	2.41 ± 1.19	1.05 ± 0.28
$SK1^{WTBM}$	DSS	0.71 ± 0.20	5.48 ± 1.34	2.83 ± 0.76	2.92 ± 1.20	1.29 ±0.35

Table S1. Blood Sphingolipid Measurements.
