# Supplementary material for: Distinct Roles for Hematopoietic and Extra-Hematopoietic Sphingosine Kinase-1 in Inflammatory Bowel Disease
Source: PLoS One. 2014 Dec 2;9(12):e113998. doi: 10.1371/journal.pone.0113998 (PMC4252067; doi:10.1371/journal.pone.0113998)
Supplement: Table S2 — Complete Blood Counts. Blood counts were analyzed following BMTP and treatment with or without DSS. Data represent mean ±SD; n≥6 mice per group. # p<0.01 compared to untreated, strain-matched control. Regular text refers to the host genotype and the superscript to the bone marrow genotype. (PDF) [file pone.0113998.s004.pdf]

| Strain | Treatment | White Blood Cells | Red Blood Cells | Platelets | Lymphocytes |
|---|---|---|---|---|---|
| **WT$^{WTBM}$** | **Water** | 3.36 ± 0.86 | 9.36 ± 0.23 | 843.80 ± 186.92 | 2688.83 ± 784.39 |
| **WT$^{WTBM}$** | **DSS** | 5.20 ± 1.65 | 9.16 ± 0.23 | 836.86 ± 6.12 | 3231.14 ± 1498.19 |
| **WT$^{SK1BM}$** | **Water** | 4.55 ± 1.02 | 9.49 ± 0.40 | 905.12 ± 125.18 | 3031.25 ± 1147.96 |
| **WT$^{SK1BM}$** | **DSS** | 9.34 ± 4.15[#] | 8.69 ± 1.23 | 804.22 ± 174.38 | 7718.11 ± 3754.78[#] |
| **SK1$^{SK1BM}$** | **Water** | 2.83 ± 0.70 | 9.13 ± 0.29 | 923.50 ± 64.44 | 2443.33 ± 556.65 |
| **SK1$^{SK1BM}$** | **DSS** | 6.22 ±5.72 | 8.31 ± 0.79 | 973.50 ± 71.04 | 4914.83 ± 4954.26 |
| **SK1$^{WTBM}$** | **Water** | 4.00 ± 1.50 | 9.46 ± 0.60 | 949.38 ± 134.46 | 3031.25 ± 1147.96 |
| **SK1$^{WTBM}$** | **DSS** | 7.32 ± 2.72 | 8.37 ± 0.62[#] | 985.20 ± 168.80 | 4256.70 ± 1584.18 |

**Table S2. Complete Blood Counts.**
