# Supplementary material for: Distinct Roles for Hematopoietic and Extra-Hematopoietic Sphingosine Kinase-1 in Inflammatory Bowel Disease
Source: PLoS One. 2014 Dec 2;9(12):e113998. doi: 10.1371/journal.pone.0113998 (PMC4252067; doi:10.1371/journal.pone.0113998)
Supplement: Table S3 — IHC analysis of bone marrow transplant mice treated with DSS. Semi-quantitative analysis of COX2, phopsho-STAT3, macrophage (F4/80), and neutrophil (Gr-1) staining in colon tissues of bone marrow transplanted tissues following DSS treatment. Regular text refers to the host genotype and the superscript to the bone marrow genotype. (PDF) [file pone.0113998.s005.pdf]

Strain	COX2	p-STAT3	Macrophages	Neutrophils
WT^{WTBM}	++++	+++	++	++
WT^{SK1BM}	+++	+	+	+
$SK1^{SK1BM}$	+	+	++	+
$SK1^{WTBM}$	++	++	+	+

Table S3. IHC analysis of bone marrow transplant mice treated with DSS.
